# Rapid detection of Tulipalin A with SESI-Orbitrap MS: an exploration across spring flowers

**DOI:** 10.1186/s13007-025-01331-6

**Published:** 2025-02-05

**Authors:** An N. T. Phan, Roy Eerlings, Hendrik G. Mengers, Lars M. Blank

**Affiliations:** Institute of Applied Microbiology - iAMB, Aachener Biology and Biotechnology - ABBt, RWTH Aachen University, Aachen, Germany

**Keywords:** Spring flower, Tulipalin, Allergic contact dermatitis, *Tulipa*, Volatile Organic compounds, Plant defense, Real-life analytics

## Abstract

**Background:**

Allergic contact dermatitis and chronic actinic dermatitis are frequently observed among florists and gardeners due to exposure to potentially allergenic plants and plant products. Tulipalin A, an alpha-methylene-gamma-butyrolactone, is a common allergen synthesized by *Tulipa* genera, but its natural occurrence across *Plantae* remains unexplored.

**Results:**

Here, we demonstrated the secondary electrospray ionization coupled Orbitrap mass spectrometry (SESI-Orbitrap MS) methodology for quantifying tulipalin A release from plants upon injury. By outlining temperature treatment, homogenization strategies and plant organ distribution, we show that processing flower samples stored at room temperature using a garlic press yielded the highest tulipalin A release upon injury. Via real-time monitoring, tulipalin A release was demonstrated to occur immediately upon homogenization. Next, the biosynthesis of tulipalin A across spring flowers was landscaped. Highlighting *Rosa*, *Gerbera*, *Neapolitanum*, *Ranunculus*, *Othocalis*, *Muscari*, *Galanthus*, *Tulipa* and *Alstroemeria* to release detectable amounts of tulipalin A upon injury. Tulipalin A was predominantly released from the *Tulipa* and *Alstroemeria* species, both belonging to the Liliales order, as stated in previous clinical and research studies.

**Conclusions:**

In conclusion, a rapid method using the SESI-Orbitrap MS is reported to detect and track tulipalin A synthesis across plant organs and outline its cross-species distribution. Our methodology can be easily adapted for mapping other volatile plant defense metabolites and identify potentially allergenic plants. By addressing these aspects, we can ensure a safer work environment for florists and gardeners.

**Supplementary Information:**

The online version contains supplementary material available at 10.1186/s13007-025-01331-6.

## Background

*Alstroemeria* and *Tulipa* cultivars, members of the *Liliaceae* order, are noteworthy cut flower species in the Netherlands [1, 2]. Incidences of allergic contact dermatitis associated with these plants have been documented among florists [3–5] and gardeners [6]. Thiboutot et al. determined a prevalence of 26% for allergic contact dermatitis among florists, with 50% exhibiting positive reactions to patch tests involving either *Alstroemeria* or *Tulipa* [7]. Although allergic contact dermatitis typically results from direct contact with damaged floral parts, a condition known as ‘tulip fingers’, airborne contact dermatitis cases have also been observed [2]. The causative agent in these plant dermatoses has been identified as tulipalin A, an antimicrobial volatile plant defense metabolite [5]. For the tulip to prevent self-injury, tulipalin A (α-methylene-γ-butyrolactone) is stored as tuliposide A (6-(4-Hydroxy-2-methylenebutyryl)-D-glucopyranose) within plant vacuoles [8]. Moreover, the compartmentalization of the lactone-forming carboxylesterases, known as tuliposide-converting enzymes, in plastids, responsible for catalyzing the intramolecular transesterification, serves as an additional protective barrier. Upon damage or infection, both tuliposide A and tuliposide-converting enzymes are released from vacuoles and plastids respectively, enabling the lactonization of tuliposide A into tulipalin A [9]. Tuliposide A constitutes 0.2–2% of tulip fresh weight and is stored across the different plant organs while tulipalin A is barely detectable [10].

Although Tulipalin A is the primary cause of allergic contact dermatitis linked with *Alstroemeria* and *Tulipa*, little is known about its mechanism of action in both humans and microbes. A chemical tolerance ranging between 0.1 and 0.3 mM for tulipalin A was observed for *Escherichia coli.* Inhibition of MurA, a key-enzyme involved in the bacterial cell wall peptidoglycan synthesis, was observed and suggested as the source of its antibacterial properties [11]. Exploring the antifungal properties of tuliposides and tulipalins on the tulip fire causing fungus, *Botrytis tulipae*, showed a tolerance up to 2.5 mM that is tenfold higher compared to bacteria. Conversion of tuliposide A into its hydroxyacid is a suggested resistance mechanism for this fungus, explaining its increased tolerance. Within the same study, a role as phytoanticipins was suggested as tulipalin presence resulted in pigmental changes of *Fusarium oxysporum* and *Gibberella zeae* [12]. Furthermore, investigation of the influence of tulipalin A on the human immune system was performed using a combination of proteomics, metabolomics and cell cycle analysis on the Jurkat T cells and THP-1 monocytes. An accumulation of chaperone proteins and changes in purine synthesis was observed when these immune cells were treated with tulipalin A [13]. Taken together, these studies in microbes and human cell culture provide the stepping stones for in-depth molecular studies to deepen our understanding of the antimicrobial and inflammatory mechanism of action of tulipalin A.

Like *Tulipa*, plants like *Crocuses* [14], *Hyacinthus* [15], and *Narcissus* [16] have also been associated with phytodermatitis. In these cases, the immune response has been associated with the needle-shaped calcium oxalate crystals, called raphides [17]. Given the similarity of symptoms across different plant-induced dermatitis cases and the unidentified nature of many plant allergens, pinpointing the specific antigen is challenging. Mapping the prevalence of well-known allergenic plant metabolites, including tulipalin A, is essential for public health [18], understanding environmental allergen impact [19, 20], and developing effective allergy prevention and management strategies [21].

To outline the distribution of plant allergens accurately, it is necessary to know threshold levels that can be used to assess their allergenicity [22]. In the case of tulipalin A, quantification is successful across tulip organs upon injury via HPLC and GC measurements [23, 24]. However, these sensitive techniques require extensive sample preparation that limits their throughput [25] and cannot provide insights into tulipalin A release and accumulation in real-time [26], essential traits to assert kinetics for mapping tulipalin A across genera. In the absence of cross-species plant-derived allergens landscapes, the list of plants reported to cause dermatitis keeps on expanding as a preventive measure.

The state-of-the-art sensitive secondary electrospray ionization coupled Orbitrap mass spectrometry system (SESI-Orbitrap MS) has emerged as a leading methodology for real-time monitoring of (semi-) volatile organic compounds (VOCs), primarily utilized in the medical field for breath gas analysis [27]. Recently, the SESI-Orbitrap MS method has demonstrated its high effectiveness in analyzing volatile organic compounds (VOCs) in diverse applications, extending beyond medical fields to include food science [28] and microbial fermentation [29]. To study the volatilome of the samples of interest, the analyte gas stream is blended with protonated water clusters generated by nano-electrospray [30]. This soft ionization step ensures minimal fragmentation [31]. Subsequently, the ionized VOCs within the sample are transferred to the Orbitrap MS for detailed analysis. The integration of nano-electrospray with high-resolution Orbitrap MS eliminates the need for separation typically associated with conventional GC-MS, enabling efficient online monitoring, even of thermolabile molecules. Moreover, unlike the hard ionization method commonly used in GC-MS, the SESI-Orbitrap MS method combines a soft ionization source with a high-resolution mass spectrometry, allowing the identification of unknown VOCs up to their molecular formula. In this study, a sensitive SESI-Orbitrap MS procedure is developed to online monitor tulipalin A release across plant organs and genera with minimal sample preparation.

## Methods

### Preparation of standards and flower samples

Commercial Tulipalin A (CAS: 547-65-9, Sigma-Aldrich, Missouri, United States) was used as standard.

To study the tulipalin A release from flower species upon injury, various sample processing conditions and the influence of temperature were assessed. For every experiment, approximately 2 g of samples were determined prior to the measurement. The flowers were cut vertically for all experiments using flower compartment. Technical replicates were conducted by measuring the same sample 3 times every 0.3 s.

For evaluating sample processing conditions, traditional milling using pestle and mortar was compared to instantaneous tissue homogenization via a garlic press. Prior to the sample processing, baselines were established by monitoring the tulipalin A signal intensity for 30 s. Next, samples were either homogenized in a single tissue crushing manner using the garlic press [28] or ground for 10 s with the pestle and mortar. Tulipalin A signal intensity was monitored prior, during and after tissue homogenization.

The influence of temperature on tulipalin A release was studied by storing the flower at -80 °C overnight, boiling of the flower by immersion for 5 min in boiling water or storage at room temperature for the duration of the experiment. Immediately after boiling or upon removal from the − 80 °C freezer, the flower organs were dissected and homogenized for tulipalin A quantification using the garlic press.

All flower species (Table 1) were purchased from and identified by a local florist (Aachen, Germany). Except for evaluating the influence of temperature on tulipalin A release, flowers were utilized on the same day of purchase.

**Table 1:** Overview of the flower species studied for their tulipalin a release potential. A single flower was obtained from every species for analysis

Flower species
*Tulipa gesneriana L.*
*Muscari botryoides*
*Alstromeria aurea*
*Hyacinthus orientalis*
*Gerbera jamesonii*
*Galanthus elwesii*
*Allium nigrum*
*Allium neapolitanum*
*Rosa luciae*
*Viola x wittrockiana*
*Crocus sativus*
*Narcissus tazetta*
*Narcissus jonquilla*
*Lamium hybridum*
*Othocalis siberica*
*Ranunculus bullatus*
*Bellis sylvestril*

For all spring flowers analyzed in this study, the phylogenetic tree was build using the web-based tool NCBI Taxonomy [32]. The flower Latin names (Table 1) were used as input to build the phylogenetic tree under the “Common Tree” setting.

### Tulipalin A analysis with SESI-Orbitrap mass spectrometry

A Super-SESI unit (Fossiliontech, Madrid, Spain) was coupled to a Q Exactive Orbitrap mass spectrometer (Thermo Fisher Scientific, Bremen, Germany). The nano-electrospray emitters Sharp Singularity (Fossiliontech) were used for all measurements. The method was used with detail parameters shown in Table S1 [28, 29]. Except stated otherwise, all experiments were run in full scan mode. The scan range was set to 50–500 *m/z* with a resolution of 70,000 and with 5 or 10 microscans. External mass calibration was performed regularly using lock masses (Table S2) to keep the mass shift of the machine between 2 and 3 ppm.

For all measurements, the SESI-Orbitrap MS was set in suction mode by setting the auxiliary gas to 0 a. u., which resulted in an air intake of approximately 800 mL/min. A glass funnel was installed in front of the SESI allowing a consistent distance [28]. After measuring the background for 0.5 min, flower samples were crushed inside the funnel with mortar and pestle or garlic press. Then, the samples were measured continuously for 1–5 min. To avoid carryover, experimental equipment was washed with methanol and gloves were changed after every measurement.

### Data treatment and analysis

The MS data treatment follows the same protocol as described by Mengers et al. [28, 29]. In short, the data were generated from SESI-Orbitrap MS using Xcalibur software version 4.2.47 (Thermo Fisher Scientific, Bremen, Germany). The raw files were converted to Excel format, providing the intensity over time profile for each feature using the open-source software MZmine2 [33]. While using the software MZmine2, the following data processing steps were applied: scan-to-scan filtering with a Savitzky-Golay filter (5 data points), mass detection (noise cut-off 1E04), and ADAP-chromatogram builder (minimum group size 30 scans, minimum intensity 1E04). The Excel files were post-processed with a self-written Python script, which sorted the measured features in noise and sample derived based on their relative intensity compared to the background.

For each sample, Student’s t-test was utilized to compare the tulipalin A signals and the background signal with 10 technical replicates (*n* = 10). The test was performed in Excel.

## Results

### Tracking Tulipalin A generation within *Tulipa gesneriana*

**Fig. 1:**
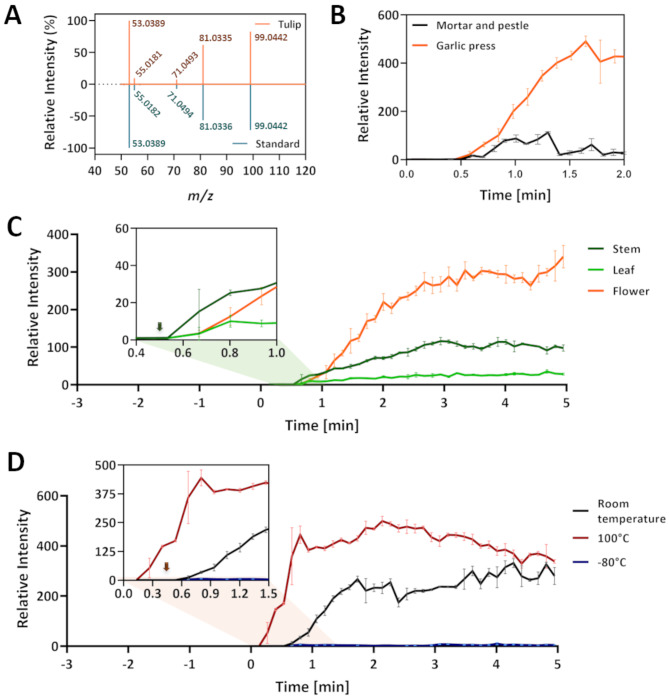
Real-time detection of Tulipalin A in *Tulipa* using a secondary electrospray ionization (SESI) coupled Orbitrap mass spectrometer (SESI-Orbitrap MS). (**A**) MS/MS mass spectrum comparison between tulipalin A from tulip flower and commercial standard. The data was normalized to the intensity of highest detected *m/z* ratio. (**B**) Release of Tulipalin A from tulip flowers upon crushing using pestle and mortar or a garlic press. (**C**) Monitoring tulipalin A accumulation from various tulip organs over time upon crushing with a garlic press (*n* = 3). (**D**) Characterization of temperature influence on tulipalin A detection. The samples were hold in front of the SESI-Orbitrap MS from 0 min to 0.5 min to establish the baseline. Then, the plant material was crushed (arrows) with a garlic press to monitor tulipalin A signal intensity overtime (*n* = 3). All data was normalized to the average of background signal measured within 0.5 min. The snippets of the real-time measurement in Fig. 1C and D showcase the moment tulipalin A signal was captured since the samples were crushed

Given the volatile nature of tulipalin A, the SESI-Orbitrap MS has the potential to detect the tulipalin A signal upon injuring of flower tissue. Analysis of the crushed flower in positive ion mode revealed a dominant ion at *m/z* 99.0442. Subsequent MS/MS analysis confirmed, through comparison with a commercial standard, that *m/z* 99.0442 represented the precursor ion [M-H]^+^ of tulipalin A (Fig. 1A).

After showcasing successful detection and quantification of tulipalin A with the SESI-Orbitrap MS, its ability to monitor the real-time accumulation of tulipalin A upon injury was investigated. Two different injuring techniques were explored to achieve stable signals (Fig. 1B). Typical crushing using pestle and mortar yielded fluctuating signal intensities. On the contrary, squashing tulip flowers with a garlic press, generated high and stable signals. This outcome was likely due to the instantaneous, homogenic tissue disruption ability of a garlic press, which was the sample squashing method adopted in subsequent experiments.

Once the sample processing method was established, characterization of the tulipalin A levels across different tulip organs was mapped. Our findings from a single (Fig. 1C) as well as from multiple tulip flowers (Figure S1) highlight the highest concentration in the flowers. Intriguingly, the tulipalin A signal in all tissue types manifested approximately 5 s after crushing, coinciding with the time it takes for the analytes to traverse the SESI-Orbitrap MS sampling inlet and reach the detector (Fig. 1C). This suggests immediate conversion of tuliposide A into tulipalin A upon tissue injury.

Next, the impact of the most common stressor, temperature, on tulipalin A generation was explored (Fig. 1D). For this, the tulip flower was immersed in boiling water for 5 min, frozen overnight at -80 °C or maintained at room temperature. Immediate release of tulipalin A was detected for the boiled tulip flower, even before being crushed. In contrast to the boiled tulip flower, the frozen tulip flower released almost no tulipalin A signal even when crushed.

### Tulipalin a release across spring flowers

**Fig. 2:**
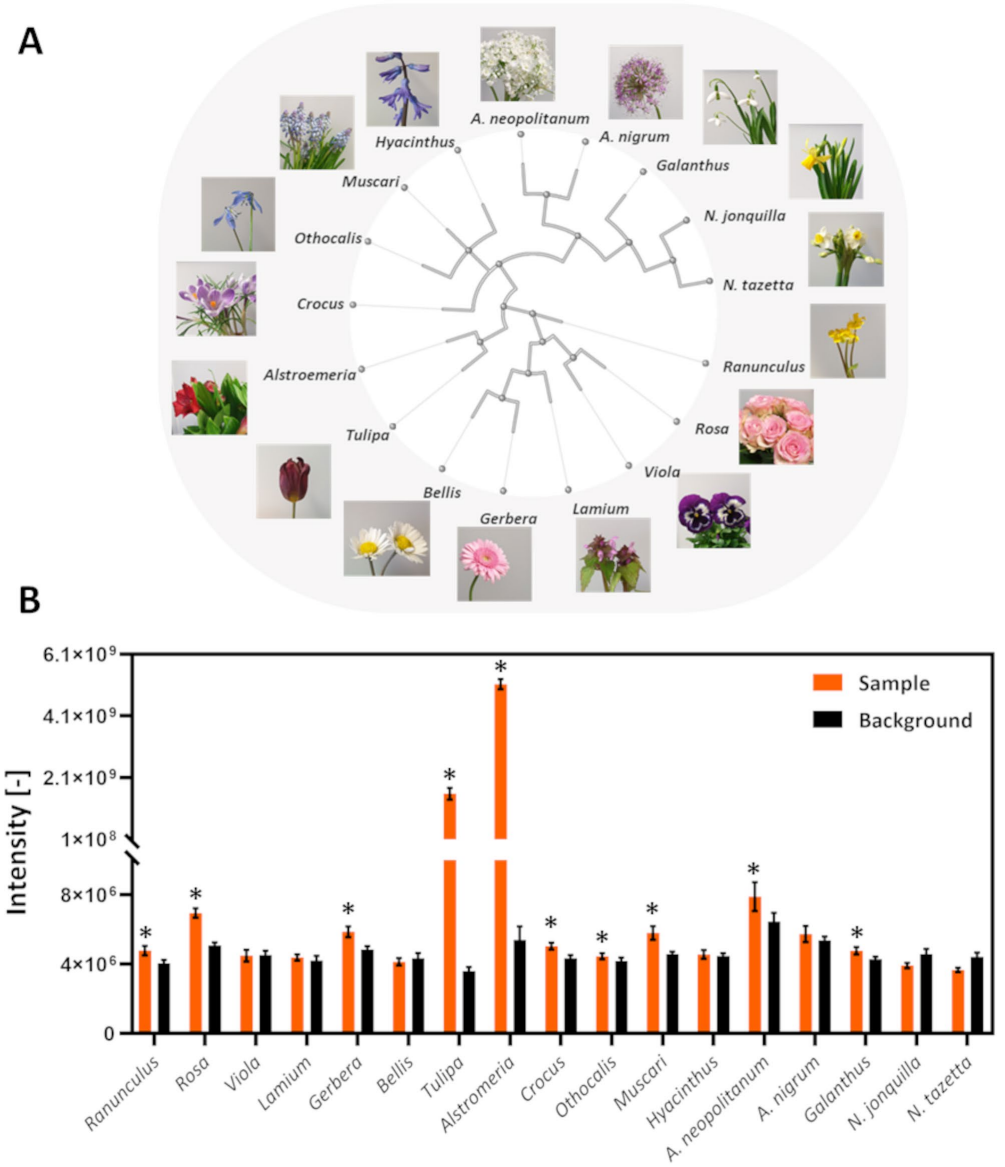
Mapping tulipalin A release across spring flowers (**A**) Simplified spring flower phylogenetic tree describing the evolutionary relationship of the flowers examined in this study. The phylogenetic tree was built using NCBI Taxonomy [32]. (**B**) Quantification of tulipalin A release across spring flower species highlighted in (**A**) upon crushing with a garlic press with SESI-Orbitrap MS. For every flower species, a background measurement was performed by holding the samples in front of the SESI-Orbitrap MS for 30 s prior to sample homogenization. (*) Samples showed significantly higher tulipalin A signal intensities compared to background using Student’s t-test (*p*-value > 0.05, *n* = 10). The error bars indicated standard deviation. All data were normalized to sample weights

Having thoroughly characterized the tulipalin A release from tulips, the prevalence of tulipalin A across various spring flowers was outlined (Fig. 2A). Phylogenetic distant species were selected to provide insights into tulipalin A biosynthesis potential across distinct clades. To evaluate potential tulipalin A accumulation upon injury, the flower species were maintained at room temperature instead of being immersed for 5 min in boiling water. Although higher maximal signal intensities are achieved when boiling the samples, immediate release of tulipalin A is observed prior to being crushed. When limited tuliposide A is present or the enzyme variant within the flower of interest is temperature sensitive, this pre-boiling step can influence the metabolic conversion or provide elevated baseline measurements thus impairing proper conclusions. The analysis revealed 10 out of 17 flower species to release a significantly higher tulipalin A signal upon crushing compared to the background (Fig. 2B). Figure S2 displays examples of full equipment spectra from samples detected at low, medium, and high signal intensities. Notably, tulip (*Tulipa*) and Peruvian lily (*Alstroemeria*) displayed the highest levels of tulipalin A, underscoring the specificity of this defense mechanism within the Liliales order [34].

## Discussion

In this study, a robust, user-friendly method using the SESI-Orbitrap MS was established to monitor release and accumulation of tulipalin A in real-time (Fig. 1A). This method sheds light on the dynamics of tulip biochemistry under various conditions. During experiments using commercial standards, we found that tulipalin A ionized easily and could be detected on our gloves or equipment if they were not properly washed. Therefore, proper cleaning between each step is necessary to avoid carryover signals. Additionally, ion suppression in the SESI source and ion competition in the C-trap are critical considerations when using SESI-Orbitrap MS [35]. Researchers interested in non-targeted metabolomics studies of tissues containing tulipalin A must employ proper strategies to avoid ion suppression.

Traditional homogenization via pestle and mortar shows to be less efficient in VOC release compared to the garlic press both in terms of total and constant tulipalin A accumulation (Fig. 1B). The garlic press not only provides a more stable release of tulipalin A across processed tissues but also yields fourfold higher concentrations compared to the fluctuating levels observed with traditional pestle and mortar milling. Comprehensive studies are imperative to clarify the variation in tissue homogenization mechanism.

Our SESI-Orbitrap MS method reveals the tulipalin A distribution across plant organs, with flowers being the primary source, followed by stem and leaves (Fig. 1C and S1). Similar results acquired from different biological samples indicates the reproducibility of our method. These findings align with both a recent enzymatic study and the transcriptomic analyses by Nomura et al. showing a predominance of the tuliposide A converting enzyme in the petals and bulb [8, 23]. A detailed examination of flower compartments, including pistils, perianth, and stamen, will provide a complete tulipalin A distribution profile.

Enforcing stress on the tulip flower sheds light on the influence of tulipalin A release upon for instance thermal shifts (Fig. 1D). The immediate detection of tulipalin A before tissue homogenization from boiled tulip flowers implies that the tulipalin A release did not originate from enzymatic reactions but rather from the thermal decomposition of tuliposide A. In the study of Kato et al., a significant reduction in tuliposide-converting enzyme activity was observed when changing the incubation temperature from 30 °C to 60 °C [36]. Interestingly, snap freezing plant material prior to the analysis eliminates the majority of the volatilome. This can result from cellular damage due to dehydration, enabling tulipalin A generation before the measurement [37]. Another hypothesis entitles the preservation of tissues in a frozen state after crushing inactivates the tuliposide-converting enzyme, preventing the initiation of the intramolecular transesterification reaction. In-depth biochemical analysis via in vitro enzymatic assays of purified tuliposide-converting enzyme will validate the second hypothesis. Furthermore, using transgenic plant models with biosynthetic traits, such as fluorescence or luminescence, responding to cellular damage or conducting transcriptomic analysis can elucidate cellular responses due to snap freezing.

Finally, landscaping tulipalin A release from spring flowers highlights that 10 out of 17 plants biosynthesize tulipalin A (Fig. 2). In agreement with previous clinical and research studies, Tulipalin A is predominantly released upon injury of *Tulipa* and *Alstroemeria* species, both belonging to the Liliales order. Contrary to expectations, both edible flowers including *Rosa*, *Gerbera*, and *Neapolitanum* and poisonous plants *Ranunculus*, *Othocalis*, *Muscari*, and *Galanthus* release significant levels of tulipalin A upon injury. This concludes edibility of a flower to not correlate with tulipalin A biosynthesizing capabilities. Despite significant release across these flowers, limited signal intensities of tulipalin A are detected, likely below the disease phenotype threshold. Alternative defense mechanisms, such as the formation of raphides, are anticipated for these spring flowers. The species diversity analyzed here underscores tulipalin A biosynthesis within the Liliales order.

Taken together, these findings enhance our knowledge of allergenic plant-derived chemical compounds. Our method can be extended towards plant volatile metabolites in general, outlining their distribution among plant organs as well as mapping their prevalence across species. Such knowledge facilitates the development of targeted interventions for the prevention and management of plant-derived allergies.

## Conclusion

In conclusion, our study demonstrates that secondary electrospray ionization coupled Orbitrap mass spectrometry (SESI-Orbitrap MS) facilitates detection and tracking of tulipalin A release across plant organs and outline its cross-species distribution. For this, we assessed parameters including temperature treatment, homogenization strategies and plant organ distribution on tulipalin A release stating the predominance of tulipalin A biosynthesis to be released from the flowers upon injury using a garlic press. By examining tulipalin A release from up to 17 distinct spring flowers, we noticed a significant tulipalin A release from *Rosa*, *Gerbera*, *Neapolitanum*, *Ranunculus*, *Othocalis*, *Muscari*, *Galanthus*, *Tulipa* and *Alstroemeria.* Tulipalin A was predominantly released from the *Tulipa* and *Alstroemeria* species, both belonging to the Liliales order, as stated in previous clinical and research studies. Our findings highlight the significance of the SESI-Orbitrap MS methodology in mapping tulipalin A release across plant organs and species. This methodology contributes to the ongoing discourse in identifying potentially allergic plant defense metabolites and offers a foundation for future advancements in landscaping volatile organic plant-derived compounds. By addressing these aspects, we can help ensure a safer work environment for florists and gardeners.

## Electronic supplementary material

Below is the link to the electronic supplementary material.

Supplementary Material 1

## Data Availability

The datasets used and/or analysed during the current study are available from the corresponding author on reasonable request.
