# Supplementary material for: Rapid detection of Tulipalin A with SESI-Orbitrap MS: an exploration across spring flowers
Source: Plant Methods. 2025 Feb 5;21:14. doi: 10.1186/s13007-025-01331-6 (PMC11795999; doi:10.1186/s13007-025-01331-6)
Supplement: Supplementary file 1 — Supplementary Material 1 [file 13007_2025_1331_MOESM1_ESM.docx]

# Supplemental information

**Table S1: Settings used for the experiments, if not otherwise mentioned**

| **Section** | **Property** | **Value** |
| --- | --- | --- |
| Method | Resolution | 70,000 |
|  | AGC target | 1E06 |
|  | Maximum IT | Auto |
|  | Spectrum data type | Profile |
| Tune file | Sheath gas flow rate | 10 a. u. |
|  | Aux gas flow rate | 0 a. u. |
|  | Spray voltage | 2.5 kV |
|  | Capillary temperature | 320 °C |
|  | S-lens RF level | 50 |
| SESI | Intake line temperature | 100 °C |
|  | Ionizer core temperature | 130 °C |

**Table S2: Lock masses used for the experiments**

| **MS Polarity** | **Exact Adduct Mass** | **Compound** | **Formula (excluding ionization)** |
| --- | --- | --- | --- |
| Positive | 149.0233 | phthalic anhydrate | C_8_H_4_O_3_ |
| Positive | 279.1591 | diisobutyl phthalate | C_16_H_22_O_4_ |
| Positive | 445.1200 | polysiloxane (n6) | C_24_H_36_Si_6_O_6_ |
| Positive | 158.96403 | sodium trifluoroacetate | C_2_F_3_NaO_2_ |
| Positive | 107.07027 | polypropylene glycole | C_3_H_6_O |
| Positive | 214.08963 | n-butyl benzenesulfonamide (plasticizer) | C_10_H_15_NO_2_S |

**
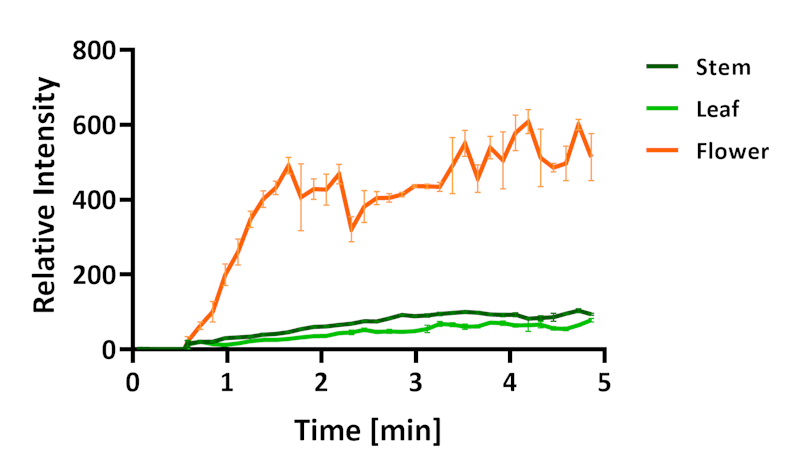
**

**Figure S1.** **Real-time detection of Tulipalin A from various tulip organs from a different flower**. The samples were hold in front of the SESI-Orbitrap MS for 0.5 mins to establish the baseline. Then, the plant material was crushed with a garlic press to monitor tulipalin A signal intensity overtime (n=3). All data was normalized to the average of background signal measured within 0.5 mins.

**
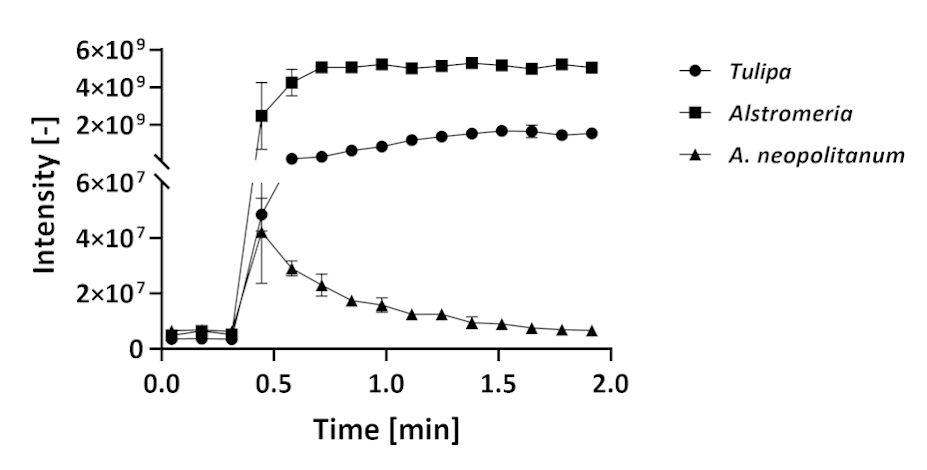
**

**Figure S2.** **Real-time detection of Tulipalin A release from flowers of *Tulipa*, *Alstromeria* and *A. neipolitanum***. The samples were hold in front of the SESI-Orbitrap MS for 0.5 mins to establish the baseline. Next, the plant material was crushed with a garlic press to monitor tulipalin A signal intensity overtime (n=3). All data was normalized to the average of background signal measured within 0.5 mins.
